# Essential Roles of Ribonucleotide Reductases under DNA Damage and Replication Stresses in Cryptococcus neoformans

**DOI:** 10.1128/spectrum.01044-22

**Published:** 2022-06-23

**Authors:** Kwang-Woo Jung, Sunhak Kwon, Jong-Hyun Jung, Yong-Sun Bahn

**Affiliations:** a Advanced Radiation Technology Institute, Korea Atomic Energy Research Institute, Jeongeup, Republic of Korea; b Department of Radiation Science and Technology, University of Science and Technology, Daejeon, Republic of Korea; c Department of Biotechnology, College of Life Science and Biotechnology, Yonsei University, Seoul, Republic of Korea; Universidade de Sao Paulo

**Keywords:** ribonucleotide reductase, DNA replication stress, DNA damage stress, *Cryptococcus neoformans*

## Abstract

A balance in the deoxyribonucleotide (dNTPs) intracellular concentration is critical for the DNA replication and repair processes. In the model yeast Saccharomyces cerevisiae, the Mec1-Rad53-Dun1 kinase cascade mainly regulates the ribonucleotide reductase (RNR) gene expression during DNA replication and DNA damage stress. However, the RNR regulatory mechanisms in basidiomycete fungi during DNA replication and damage stress remain elusive. Here, we observed that in C. neoformans*, RNR1* (large RNR subunit) and *RNR21* (one small RNR subunit) were required for cell viability, but not *RNR22* (another small RNR subunit). *RNR22* overexpression compensated for the lethality of *RNR21* suppression. In contrast to the regulatory mechanisms of RNRs in S. cerevisiae, Rad53 and Chk1 kinases cooperatively or divergently controlled *RNR1* and *RNR21* expression under DNA damage and DNA replication stress. In particular, this study revealed that Chk1 mainly regulated *RNR1* expression during DNA replication stress, whereas Rad53, rather than Chk1, played a significant role in controlling the expression of *RNR21* during DNA damage stress. Furthermore, the expression of *RNR22*, not but *RNR1* and *RNR21*, was suppressed by the Ssn6-Tup1 complex during DNA replication stress. Notably, we observed that *RNR1* expression was mainly regulated by Mbs1, whereas *RNR21* expression was cooperatively controlled by Mbs1 and Bdr1 as downstream factors of Rad53 and Chk1 during DNA replication and damage stress. Collectively, the regulation of RNRs in C. neoformans has both evolutionarily conserved and divergent features in DNA replication and DNA damage stress, compared with other yeasts.

**IMPORTANCE** Upon DNA replication or damage stresses, it is critical to provide proper levels of deoxynucleotide triphosphates (dNTPs) and activate DNA repair machinery. Ribonucleotide reductases (RNRs), which are composed of large and small subunits, are required for synthesizing dNTP. An imbalance in the intracellular concentration of dNTPs caused by the perturbation of RNR results in a reduction in DNA repair fidelity. Despite the importance of their roles, functions and regulations of RNR have not been elucidated in the basidiomycete fungi. In this study, we found that the roles of *RNR1*, *RNR21*, and *RNR22* genes encoding RNR subunits in the viability of C. neoformans. Furthermore, their expression levels are divergently regulated by the Rad53-Chk1 pathway and the Ssn6-Tup1 complex in response to DNA replication and damage stresses. Therefore, this study provides insight into the regulatory mechanisms of RNR genes to DNA replication and damage stresses in basidiomycete fungi.

## INTRODUCTION

Upon DNA damage stress in eukaryotic cells, the cell cycle is arrested and the DNA repair machinery is activated by increased gene expression. The repair process requires adequate deoxynucleotide triphosphate (dNTP) levels and activation of DNA repair proteins. The dNTP is synthesized from ribonucleotide triphosphate (NTP) by the reduction of the C2’-OH bond through a ribonucleotide reductase (RNR). If the intracellular concentration of dNTPs is unbalanced, DNA replication fork progress is stalled, which is called DNA replication stress (1). Therefore, during DNA replication and repair, homeostasis of intracellular levels of dNTPs, which depends on RNR regulation, is a prerequisite for living organisms.

The functions and regulatory mechanisms of RNR have been well characterized in the model yeast Saccharomyces cerevisiae. RNR consists of a large subunit, R1 and a small subunit, R2. The R1 subunits are composed of a homodimer encoded by *RNR1* and Rnr1 contains both catalytic and allosteric sites that determines the enzyme activity (2). In addition to *RNR1*, a gene encoding a large subunit *RNR3* has been identified, but it is not involved in viability, contrary to *RNR1*, which is essential for viability (3). The small R2 subunits consist of a heterodimer encoded by *RNR2* and *RNR4*. Similar to *RNR1*, both *RNR2* and *RNR4* are required for viability (4, 5). The expression of RNR genes is inducible in response to DNA damage stress, as well as the cell cycle. *RNR1* expression is regulated in a cell cycle-dependent manner and induced in response to DNA damage insults such as 4-nitroquinoline 1-oxide (4-NQO) (3). The expression levels of *RNR3* are significantly lower at the basal level, whereas those of *RNR3* are highly increased in response to DNA damage stress (3). Similar to *RNR1* and *RNR3*, the expression of *RNR2* and *RNR4* is also inducible under DNA damage-stress conditions (4, 5). Under DNA damage stress, the Crt1 transcription factor is phosphorylated in a Mec1-Rad53-Dun1-dependent manner and *RNR2*, *RNR3*, and *RNR4* expression is induced by the dissociation of Crt1 from the upstream RNR gene regions (6, 7). In addition to the transcriptional level, protein localization of Rnr2 and Rnr4 is regulated by Dif1. In the S-phase or under DNA damage stress, Rnr2 and Rnr4 translocate from the nucleus to the cytoplasm to bind to the Rnr1 complex, forming an active complex. However, Dif1 binds to the Rnr2-Rnr4 complex and relocates it to the nucleus. Under DNA damage stress, Dun1 kinase phosphorylates Dif1, which results in the degradation of Dif1 and increases the cytoplasmic localization of the Rnr2-Rnr4 complex (8). During DNA damage stress, Rad53 activates Ixr1, which contains a high-mobility group box (HMG) domain and binds to the promoter region of *RNR1* by regulating histone levels in a Dun1-independent manner (9). Following DNA damage, the Mec1-Rad53-Dun1 kinase cascade phosphorylates Sml1, an inhibitor of Rnr1, thereby degrading Sml1 (10). Collectively, these results indicate that the Mec1-Rad53-Dun1 kinase cascade is critical for the regulation of RNR gene transcription and protein activation.

In addition to S. cerevisiae, the regulatory mechanisms of RNR genes in other fungal pathogens have been studied. In Candida albicans, the large subunits of RNR are encoded by *RNR1* and *RNR3* and the small subunits of RNR are encoded by *RNR21* and *RNR22*. Similar to that in S. cerevisiae, the expression of *RNR1*, *RNR3*, and *RNR21* is induced under DNA replication stress (11, 12). In particular, the expression levels of *RNR1* and *RNR21*, but not *RNR3*, are induced by DNA replication stress via an Nrm1-dependent pathway (11). In Aspergillus nidulans, the large and small subunits of the RNR complex are encoded by *rnsA* and *rnrA*, respectively, both of whose expression is induced in the presence of DNA damage stress agents such as methyl methanesulfonate (MMS), and 4-NQO (13, 14). Moreover, the expression of these genes is redundantly controlled by CsnD/CsnE signaling and NpkA under genotoxic stress (13).

Cryptococcus neoformans is considered a pathogenic model system in basidiomycetes due to its pathogenic mechanisms, which was elucidated by genetic and molecular techniques. Recently, our group reported that C. neoformans contains an evolutionarily conserved and distinct signaling network in response to DNA damage stress. Briefly, upon DNA damage stress, Mec1 and Tel1, which are homologous to ATR and ATM, respectively, in humans and members of the phosphatidylinositide-3-kinase (PI3K) family cooperatively phosphorylate Rad53, which is homologous to CHK2 in humans. Activated Rad53 increases the expression of DNA repair genes, such as *RAD51*, through the regulation of the Bdr1 transcription factor, which is uniquely found in the Cryptococcus species complex (15–17). Chk1 kinase also cooperatively regulates DNA replication and damage stresses, but its downstream targets remain unclear (15).

Despite the critical role of RNR in ascomycete fungi, it has not been well characterized in basidiomycete fungi. To answer this question, we examined the growth requirement and expression levels of RNR genes in strains lacking genes belonging to the DNA repair pathway in response to DNA damage and replication stresses, and performed phenotypic analysis using promoter replacement strains. Here, we found that the regulation and role of RNR in C. neoformans have evolutionarily conserved and divergent features in DNA replication and DNA damage stress.

## RESULTS

### Role of RNR genes in the viability of C. neoformans.

**FIG 1:**
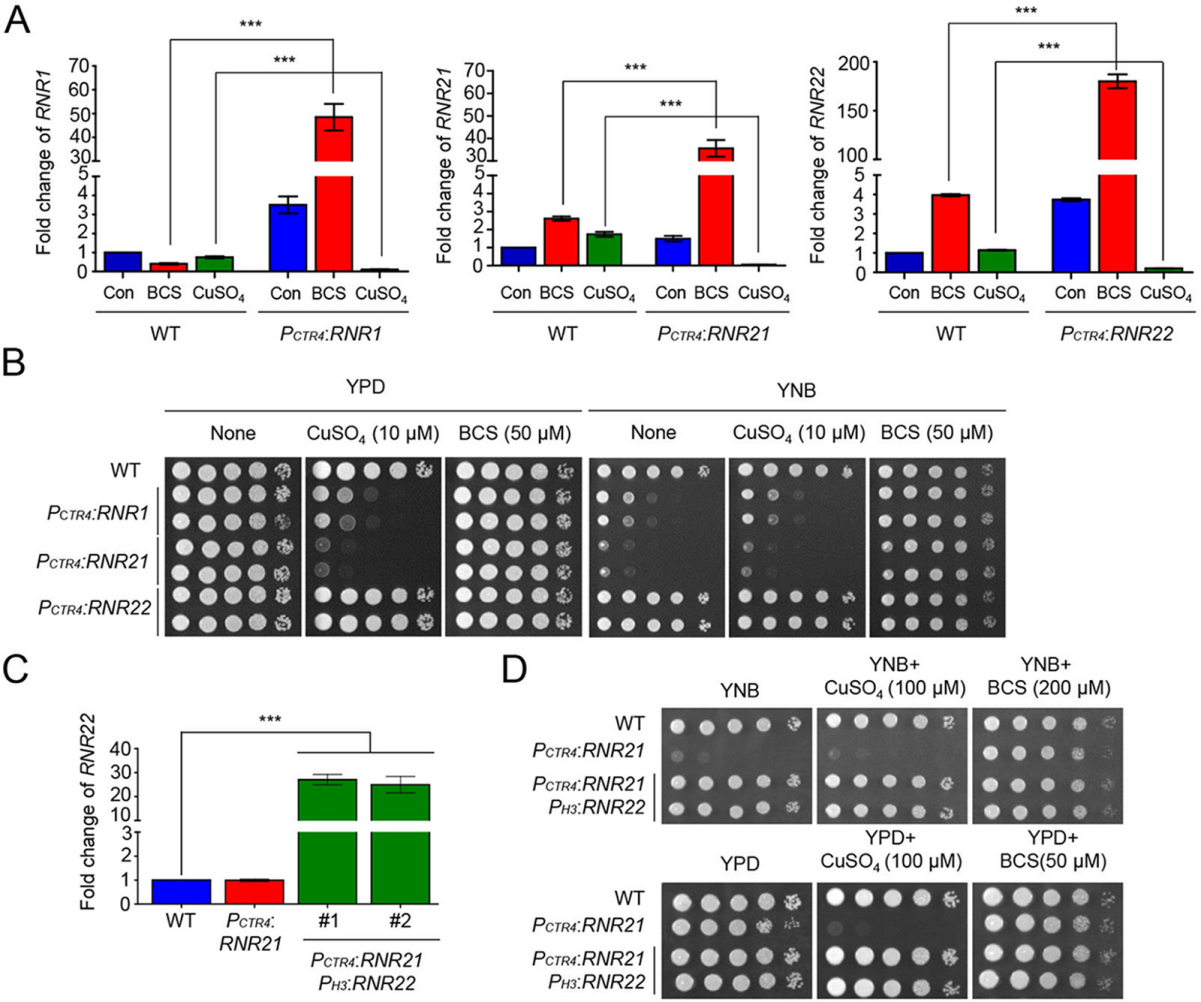
Rnr1 and Rnr21, not Rnr22, are required for viability in C. neoformans. (A) Fold change of *RNR1*, *RNR21*, and *RNR22* in *P_CTR4_*:*RNR1*, *P_CTR4_*:*RNR21*, and *P_CTR4_*:*RNR22* strains in the presence of BCS or Cu^2+^. Statistical significance of differences was determined by one-way analysis of variance (ANOVA) with Bonferroni’s test. Error bars indicate standard error of the mean (***, *P < *0.001). (B) *RNR1* and *RNR21* are required for viability. Strains (B: WT, *P_CTR4_*:*RNR1*, *P_CTR4_*:*RNR21*, and *P_CTR4_*:*RNR22* promoter replacement strains; D: WT, *P_CTR4_*:*RNR21*, and *P_CTR4_*:*RNR21 P_H3_*:*RNR22*) were cultured in a liquid YPD medium. The strains were 10-fold serially diluted and spotted onto YNB medium or YPD medium containing the indicated concentration of CuSO_4_ and BCS. Strains were further incubated at 30°C for 4 days and photographed. (C) The constitutive overexpression of *RNR22* in the *P_CTR4_*:*RNR21* strain. Total RNA was isolated from WT, *P_CTR4_*:*RNR21*, and *P_CTR4_*:*RNR21 P_H3_*:*RNR22* strains (KW1458 and KW1459) and cDNA was synthesized from these total RNA samples. Statistical significance of differences was determined by one-way analysis of variance (ANOVA) with Bonferroni’s test. Error bars indicate standard deviation (*** *P < *0.001). (D) Complementation of reduced viability through *RNR22* overexpression.

ScRNR genes, including *RNR1* and *RNR2*, and C. albicans *RNR2* are essential for viability (3, 4, 18). A previous study suggested that C. neoformans has one *RNR1* homolog, encoding a large RNR subunit, and two homologous genes, *RNR21* and *RNR22*, which encode small RNR subunits (19). To address whether *CnRNR1*, *CnRNR21*, and *CnRNR22* are required for survival, we constructed conditional *RNR1*, *RNR21*, and *RNR22* expression strains by replacing each native promoter with a copper-regulated *CTR4* promoter (*P_CTR4_*) upstream of the ATG start codon or the 5′-UTR of the *RNR1*, *RNR21* and *RNR22* genes (Fig. S1). We confirmed the correct genotype of the promoter replacement strains using Southern blot analysis (Fig. S1) and the expression of *RNR1*, *RNR21*, and *RNR22* in the presence of copper sulfate (CuSO_4_), which suppresses the *CTR4* downstream gene expression, and bathocuproine disulphonate (BCS), which strongly induces the *CTR4* downstream gene expression (20). We observed that the *RNR1*, *RNR21*, and *RNR22* expression in *P_CTR4_*:*RNR1*, *P_CTR4_*:*RNR21*, and *P_CTR4_*:*RNR22* strains were markedly increased under BCS treatment but suppressed under CuSO_4_ treatment (Fig. 1A). Next, we observed the growth changes in *P_CTR4_*:*RNR1*, *P_CTR4_*:*RNR21*, and *P_CTR4_*:*RNR22* strains in the presence of CuSO_4_ or BCS. The *P_CTR4_*:*RNR1* and *P_CTR4_*:*RNR21* strains exhibited growth defects compared with wild-type (WT) in the presence of CuSO_4_, whereas their growth defects were suppressed by BCS (Fig. 1B). In contrast, the *P_CTR4_*:*RNR22* strains displayed growth comparable to that of WT regardless of the presence of CuSO_4_ or BCS (Fig. 1B). These data indicate that *RNR1* and *RNR21*, but not *RNR22*, are required for cell viability.

In S. cerevisiae, the overexpression of *RNR3* suppresses lethality in the absence of *RNR1* (5). Because our present study found that *RNR21* and *RNR22* encode a small subunit of the RNR complex and *RNR21*, not *RNR22*, is required for viability in C. neoformans, we addressed whether *RNR22* overexpression could suppress lethality due to the loss of *RNR21*. To test this hypothesis, we overexpressed *RNR22* in the background of the *P_CTR4:_RNR21* strain by inserting the H3 constitutive promoter and confirmed *RNR22* overexpression using quantitative reverse transcription-PCR (qRT-PCR) (Fig. 1C). Similar to the compensation of *RNR3* in the lethality of *RNR1* in S. cerevisiae, the overexpression of *RNR22* suppressed the lethality caused by the reduction of *RNR21* expression (Fig. 1D). These data suggest that Rnr22 also retains the function of RNR similar to Rnr21.

### RNR genes are differentially regulated by the Rad53-Chk1 pathway and the Ssn6-Tup1 complex under DNA replication stress.

**FIG 2:**
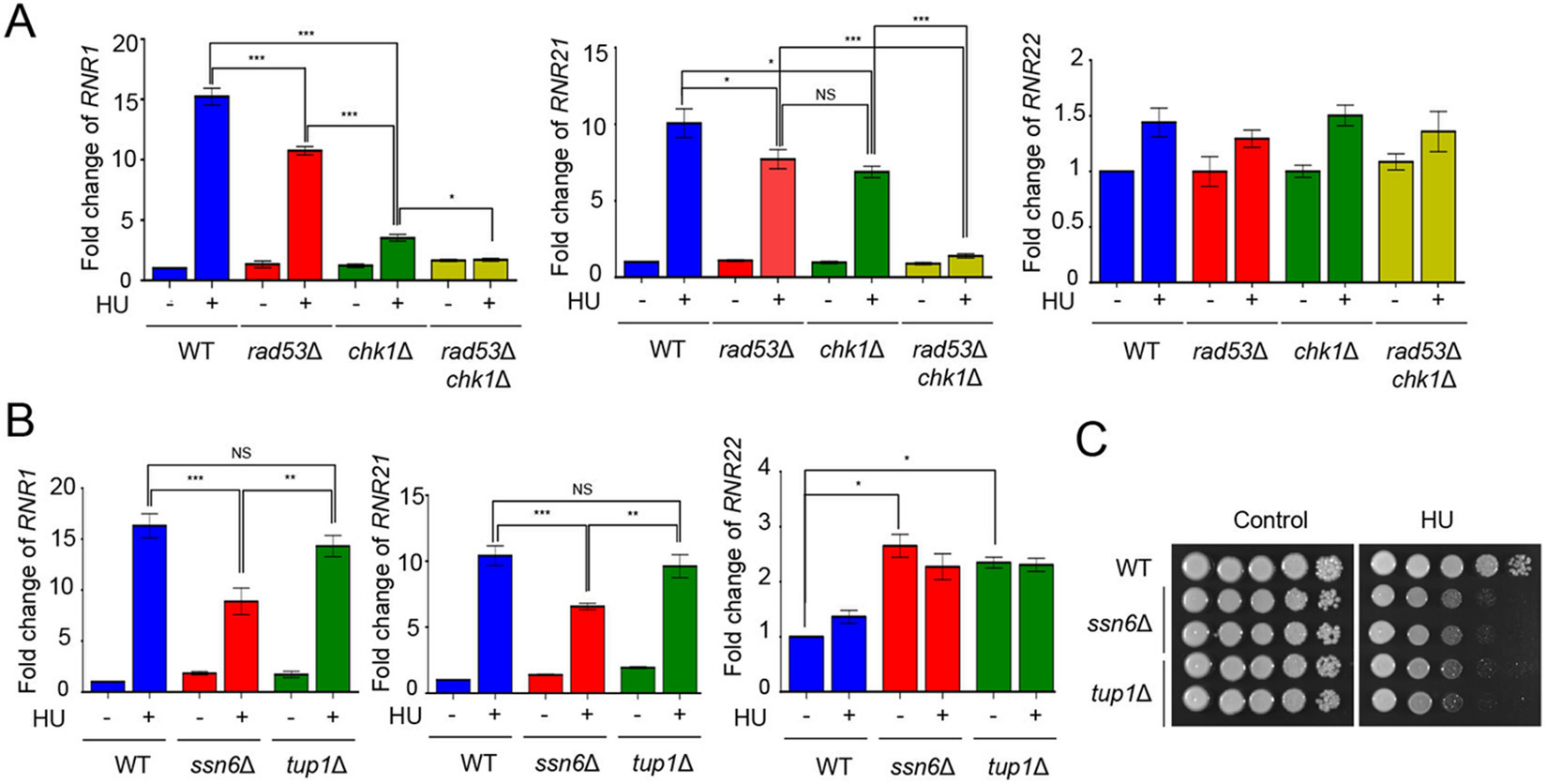
Expression levels of *RNR1*, *RNR21*, and *RNR22* after HU treatment. (A) Expression of *RNR1* and *RNR21* was regulated by both Rad53 and Chk1. (B) Ssn6-Tup1 complex suppressed *RNR22* expression. Quantitative RT-PCR analysis was performed using cDNA synthesized from the total RNA isolated from WT H99, *rad53*Δ, *chk1*Δ, *ssn6*Δ, *tup1*Δ, and *rad53*Δ *chk1*Δ double mutant treated with 50 mM HU. Three independent biological samples were analyzed with technical duplicates. Error bars indicate standard error of the mean (S. E. M). (*, *P < *0.05; **, *P < *0.01; ***, *P < *0.001; NS, nonsignificant). (C) The *ssn6*Δ and *tup1*Δ mutants were sensitive to HU. The strains were cultured in liquid yeast extract peptone dextrose (YPD) medium, which was serially diluted and spotted onto the YPD medium containing HU (50 mM). The strains were further incubated at 30°C and photographed daily.

DNA replication stress arises from diverse sources such as DNA lesion and misincorporation of ribonucleotide (1). The hydroxyurea (HU), which is an RNR inhibitor causing depletion of dNTPs, is widely used for induction of DNA replication stress (2). Given that *RNR1* and *RNR21* expression is induced in C. neoformans and that the expression levels of *RNR2*, *RNR3*, and *RNR4* are regulated by Rad53 kinases under DNA replication stress in S. cerevisiae (5, 19), we measured the expression levels of RNR genes under HU treatment in the WT and *rad53*Δ mutant strains. Unlike RNR expression patterns in S. cerevisiae, *RNR1* and *RNR21* induction levels in the *rad53*Δ mutant were slightly lower than those in the WT (Fig. 2A), indicating that another (or other) factor contributes to the regulation of *RNR1* and *RNR21* expression. We further monitored RNR expression in the strains lacking *CHK1*, which is an effector kinase in the DNA repair pathway, similar to Rad53 kinase and both *CHK1* and *RAD53*. Notably, *RNR1* induction was significantly lower in the *chk1*Δ and *rad53*Δ *chk1*Δ double mutants than that in the *rad53*Δ mutant and *RNR1* induction in the *rad53*Δ *chk1*Δ double mutant was slightly lower than that in the *chk1*Δ mutant (Fig. 2A). These data suggest that *RNR1* induction is cooperatively regulated by both Rad53 and Chk1 and Chk1 plays a major role in *RNR1* induction. Similar to *RNR1* expression, *RNR21* expression in *rad53*Δ *chk1*Δ double mutants did not change in the presence or absence of HU, whereas *RNR21* induction levels in the *chk1*Δ mutant were similar to those in the *rad53*Δ mutant (Fig. 2A). The *RNR22* induction was not observed in *rad53*Δ, *chk1*Δ, and *rad53*Δ *chk1*Δ double mutants as that in WT under HU treatment (Fig. 2A).

In S. cerevisiae, *RNR2*, *RNR3,* and *RNR4* expression is transcriptionally suppressed by the Ssn6-Tup1 complex with Crt1 transcription factor (6, 21). Therefore, we addressed whether the expression of RNR genes is regulated by the Ssn6-Tup1 complex in C. neoformans. Unlike in S. cerevisiae, the expression levels of *RNR1* and *RNR21* in the *ssn6*Δ and *tup1*Δ mutants were similar to those in the WT at the basal level (Fig. 2B). However, the induction patterns of *RNR1* and *RNR21* in the *ssn6*Δ and *tup1*Δ mutants appeared to be distinct from each other. The expression levels of *RNR1* and *RNR21* in the *tup1*Δ mutant were similar to those in the WT in the presence of HU, whereas *RNR1* and *RNR21* expression in the *ssn6*Δ mutant was induced to a lower extent compared to WT (Fig. 2B). These data indicate that Ssn6 partially contributes to the positive regulation of *RNR1* and *RNR21* expression in a Tup1-independent manner under HU treatment. However, *RNR22* expression was intrinsically induced in both the *ssn6*Δ and *tup1*Δ mutants in the absence of HU, whereas it was not further increased in the presence of HU (Fig. 2B). Because Ssn6 and Tup1 negatively control the expression levels of *RNR22*, we checked whether the *ssn6*Δ and *tup1*Δ mutants exhibited HU-resistance. Although Ssn6, but not Tup1, was required for the full induction of *RNR1* and *RNR21*, both the *ssn6*Δ and *tup1*Δ mutants exhibited increased HU sensitivity compared to the WT (Fig. 2C). Taken together, the HU-mediated induction of *RNR1* and *RNR21* was cooperatively regulated by Rad53, Chk1, and Ssn6, whereas the Ssn6-Tup1 complex controls basal *RNR22* expression in C. neoformans.

### The Mbs1 transcription factor controls the induction of *RNR1* and *RNR21* as a downstream factor of Rad53 and Chk1 under DNA replication stress.

**FIG 3:**
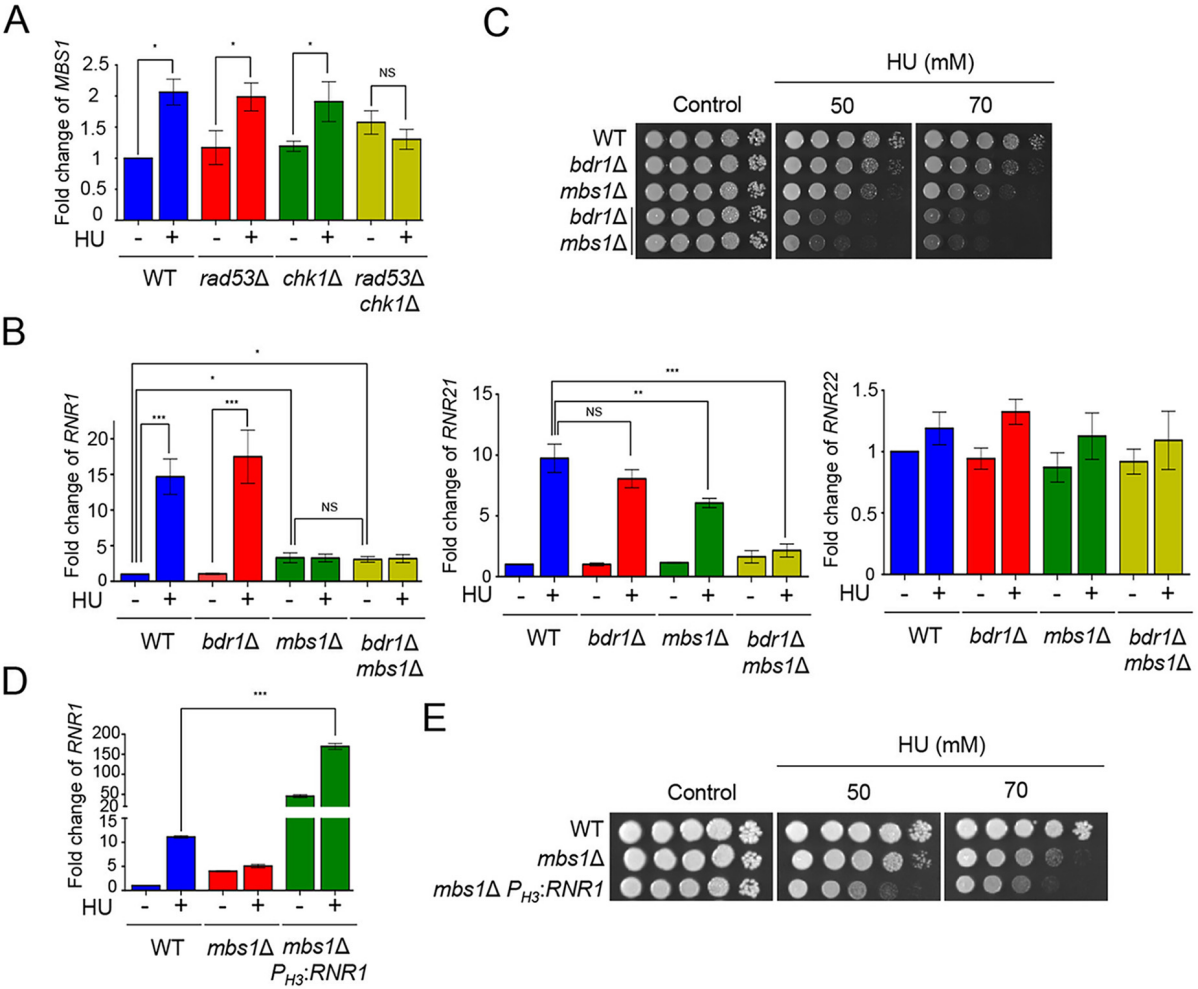
Bdr1 and Mbs1 cooperatively regulated expression levels of *RNR1* and *RNR21* genes under HU treatment. (A and B) Expression of *MBS1*, *RNR1*, *RNR21*, and *RNR22* in the signaling mutants under HU treatment. qRT-PCR analysis was performed using cDNA synthesized from total RNA isolated from WT H99, *rad53*Δ, *chk1*Δ, *rad53*Δ *chk1*Δ double mutant, *bdr1*Δ, *mbs1*Δ, and *bdr1*Δ *mbs1*Δ double mutant treated with HU 50 mM. Three independent biological samples were analyzed with duplicate technical replicates. Error bars indicate standard error of the mean (S. E. M). (*, *P < *0.05; **, *P < *0.01; ***, *P < *0.001; NS, nonsignificant). (C) The deletion of both *BDR1* and *MBS1* resulted in synergistic growth defects in response to HU. Strains were cultured in a liquid YPD medium and were serially diluted and spotted onto the YPD medium containing the indicated concentration of HU. Strains were further incubated at 30°C and photographed daily. (D) Constitutive overexpression of *RNR1* in *mbs1*Δ *P*_H3_:*RNR1* strain in the presence or absence of HU. (E) Overexpression of *RNR1* in *mbs1*Δ mutant resulted in increased HU sensitivity.

Previous studies have revealed that *RNR1* expression is regulated by the MCB binding factor (MBF) complex composed of Mbp1 and Swi6 in S. cerevisiae (22, 23). Supporting this notion, perturbation of *MBS1* (Mbp1- and Swi4-like protein 1) increases HU sensitivity in C. neoformans (24, 25). Given that *MBS1* is transcriptionally controlled in response to environmental cues (24) and *RNR1* and *RNR21* expression is regulated by Rad53 and Chk1, we measured *MBS1* expression in WT, *rad53*Δ, *chk1*Δ, and *rad53*Δ *chk1*Δ double mutants under HU treatment. *MBS1* expression in the WT, *rad53*Δ, and *chk1*Δ mutants significantly increased in the presence of HU, whereas that in the *rad53*Δ *chk1*Δ double mutants did not change (Fig. 3A). These data indicate that Mbs1 is a downstream target of Rad53 and Chk1.

Our previous studies have reported that Bdr1 is a downstream TF regulated by Rad53 (15, 16). To elucidate the regulation of *RNR1* and *RNR21* expression by Bdr1 and Mbs1 as downstream factors of Rad53 and Chk1, we constructed *bdr1*Δ *mbs1*Δ double mutants (Fig. S1) and measured the expression of these genes in the WT, *bdr1*Δ, *mbs1*Δ, and *bdr1*Δ *mbs1*Δ double mutants. *RNR1* expression in the *bdr1*Δ mutant was similar to that in the WT in the presence of HU (Fig. 3B). Notably, similar to the *RNR1* expression pattern in the *rad53*Δ *chk1*Δ double mutant, *RNR1* was not induced at all in the *mbs1*Δ mutants, although *RNR1* was intrinsically increased at the basal level (Fig. 3B). Furthermore, the expression level of *RNR1* in the *mbs1*Δ mutant was not distinguishable from that of *RNR1* in the *bdr1*Δ *mbs1*Δ double mutant in the absence or presence of HU (Fig. 3B), indicating that Mbs1, but not Bdr1, mainly controls *RNR1* induction in the presence of HU. In the case of *RNR21*, *RNR21* induction level in the *mbs1*Δ mutant, not but *bdr1*Δ mutant, slightly reduced compared with that of WT in the presence of HU. Notably, the *RNR21* induction level in response to HU was markedly reduced in the *bdr1*Δ *mbs1*Δ double mutant compared to the *bdr1*Δ and *mbs1*Δ single mutants (Fig. 3B), as shown in the *rad53*Δ *chk1*Δ double mutant. However, Bdr1 and Mbs1 were not involved in the expression levels of *RNR22* as Rad53 and Chk1 were (Fig. 3B). Collectively, Mbs1 is involved in regulating the expression levels of both *RNR1* and *RNR21*, whereas Bdr1 partly controls *RNR21* expression.

Because the present results showed that Mbs1 is critical for the regulation of *RNR1* and *RNR21* in response to HU, we performed a survival assay with *bdr1*Δ *mbs1*Δ double mutants in HU treatment. Supporting *RNR* expression in the *bdr1*Δ *mbs1*Δ double mutant, the *bdr1*Δ *mbs1*Δ double mutant showed more severe growth defect in response to HU than each single mutant (Fig. 3C).

We hypothesized that *RNR1* overexpression could restore HU resistance in the *mbs1*Δ mutant for the following reasons. First, the induction of *RNR1*, but not *RNR21*, was significantly lower in the *mbs1*Δ mutant than in the C. neoformans WT. Second, overexpression of *RNR1* increases resistance to DNA damage stress in S. cerevisiae (26). To prove this hypothesis, we constructed a constitutive *RNR1* overexpression strain in the background of the *mbs1*Δ mutant using *H3* promoter replacement (Fig. S1). We confirmed the overexpression of *RNR1* by qRT-PCR analysis using *RNR1* gene-specific primers in the presence or absence of HU (Fig. 3D). Unexpectedly, *RNR1* overexpression strains in the background of the *mbs1*Δ mutant showed growth defects compared to the *mbs1*Δ mutant in response to HU (Fig. 3E). These data indicate that other factors contribute to HU resistance in the *mbs1*Δ mutant.

### Regulation of RNR expression following DNA damage stress.

**FIG 4:**
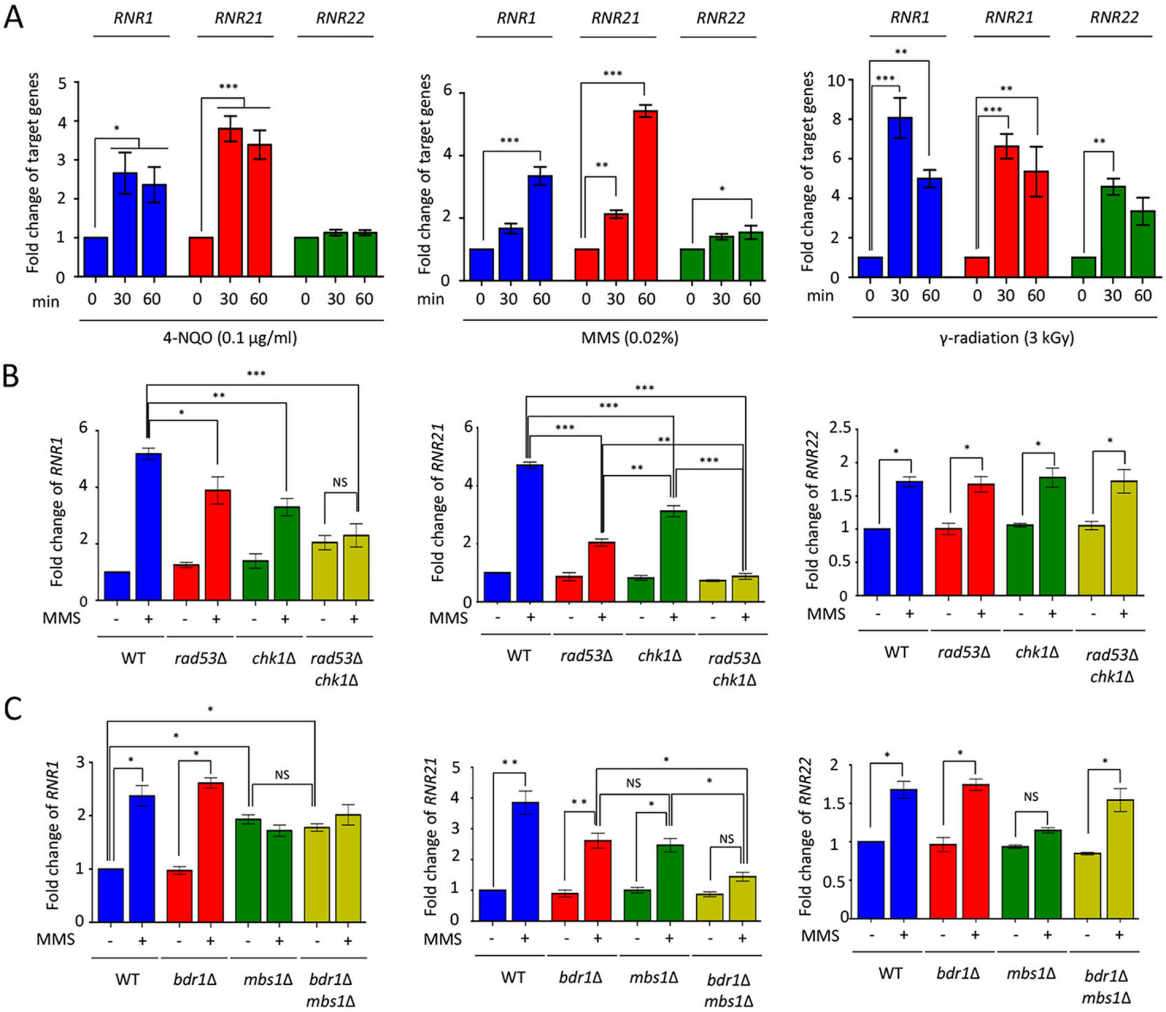
The expression levels of *RNR1*, *RNR21*, and *RNR22* genes in response to DNA damage stress. (A) Expression of *RNR1*, *RNR21*, and *RNR22* in WT upon 4-NQO or MMS treatment or gamma radiation exposure. (B and C) Expression levels of *RNR1*, *RNR21*, and *RNR22* in WT, *rad53*Δ, *chk1*Δ, *rad53*Δ *chk1*Δ double mutant, *bdr1*Δ, *mbs1*Δ, and *bdr1*Δ *mbs1*Δ double mutants under MMS treatment. The qRT-PCR analysis was performed using cDNA synthesized from total RNA isolated from WT H99, *rad53*Δ, *chk1*Δ *rad53*Δ *chk1*Δ double mutant, *bdr1*Δ, *mbs1*Δ, and *bdr1*Δ *mbs1*Δ double mutants treated with MMS 0.02%. Three independent biological samples were analyzed with duplicate technical replicates. Error bars indicate standard error of the mean (S. E. M). (*, *P < *0.05; **, *P < *0.01; ***, *P < *0.001; NS, nonsignificant).

In S. cerevisiae, RNR expression is induced in response to diverse DNA-damaging stress agents, such as MMS (an inducer of DNA alkylation) and 4-NQO (a DNA damage inducer through the production of reactive oxygen species) (4, 5, 27). We wanted to check whether the expression levels of RNR genes were altered in the presence of DNA damage insults, as shown by treatment with HU. First, we measured the expression levels of RNR genes under diverse DNA damage insults, such as MMS, 4-NQO, and gamma radiation exposure (ionizing radiation inducing diverse forms of DNA damage, such as double-strand breaks [DSB]). In the presence of MMS or 4-NQO, *RNR1* and *RNR21* expression gradually increased, whereas those of *RNR22* were induced to a lesser extent by MMS, not but 4-NQO, treatment. Interestingly, all the RNR genes were induced by gamma radiation exposure (Fig. 4A). These data suggest that the expression of RNR genes could be induced by DNA damage inducers.

Next, we investigated whether the expression patterns of *RNR1* and *RNR21* occur in Rad53- and Chk1-dependent manners under MMS treatment and radiation exposure, similar to those under the HU treatment. Unexpectedly, the expression patterns of *RNR1* and *RNR21* in MMS and radiation exposure groups appeared to be distinguishable from those under the HU treatment. After radiation exposure, the *RNR1* gene expression was induced in the *rad53*Δ, *chk1*Δ, and *rad53*Δ *chk1*Δ double mutants, but to a lesser extent than in WT. Notably, the level of *RNR21* induction in the *rad53*Δ mutant was significantly lower than that in the WT and *chk1*Δ mutant. Furthermore, *RNR21* induction levels in the *rad53*Δ mutant were similar to those in the *rad53*Δ *chk1*Δ double mutant, indicating that Chk1 did not affect in *RNR21* expression after radiation exposure (Fig. S2). Under MMS treatment, *RNR1* induction in the *rad53*Δ and *chk1*Δ mutants was slightly lower than that in the WT, whereas *RNR1* expression did not change in the *rad53*Δ *chk1*Δ double mutant in the presence or absence of MMS (Fig. 4B). The induction level of *RNR21* in the *rad53*Δ and *chk1*Δ mutants was lower than that in WT and *RNR21* expression was not increased in the *rad53*Δ *chk1*Δ double mutant after treatment with MMS. However, *RNR22* induction occurred in a Rad53- and Chk1-independent manner following MMS treatment and gamma radiation exposure (Fig. 4B; Fig. S2). Next, to further address whether the Bdr1 and Mbs1 transcription factors participate in the regulation of *RNR1*, *RNR21*, and *RNR22*, we monitored their expression levels in WT, *bdr1*Δ, *mbs1*Δ, and *bdr1*Δ *mbs1*Δ double mutants under MMS treatment. The *RNR1* expression was induced in response to MMS treatment in the *bdr1*Δ mutant like the WT, similar to the expression under HU treatment, but not in the *mbs1*Δ and *bdr1*Δ *mbs1*Δ double mutants (Fig. 4C). In the case of *RNR21*, Mbs1 and Bdr1 cooperatively regulated the expression of *RNR21* in response to MMS, as shown in the HU treatment group (Fig. 4C). Notably, MMS-mediated induction of *RNR22* expression was not observed in the *mbs1*Δ mutant. However, it was restored to WT levels in the *bdr1*Δ *mbs1*Δ double mutant, indicating that Mbs1 and Bdr1 may play opposing roles in *RNR22* regulation (Fig. 4C). Collectively, under MMS treatment-induced DNA damage, Rad53 and Chk1 cooperatively regulate the expression levels of *RNR1* and *RNR21* as shown in the HU treatment group, and Mbs1 is required for the regulation of *RNR1*, *RNR21*, and *RNR22*.

### Transcriptional perturbation of *RNR21* increases DNA replication and damage stresses.

**FIG 5:**
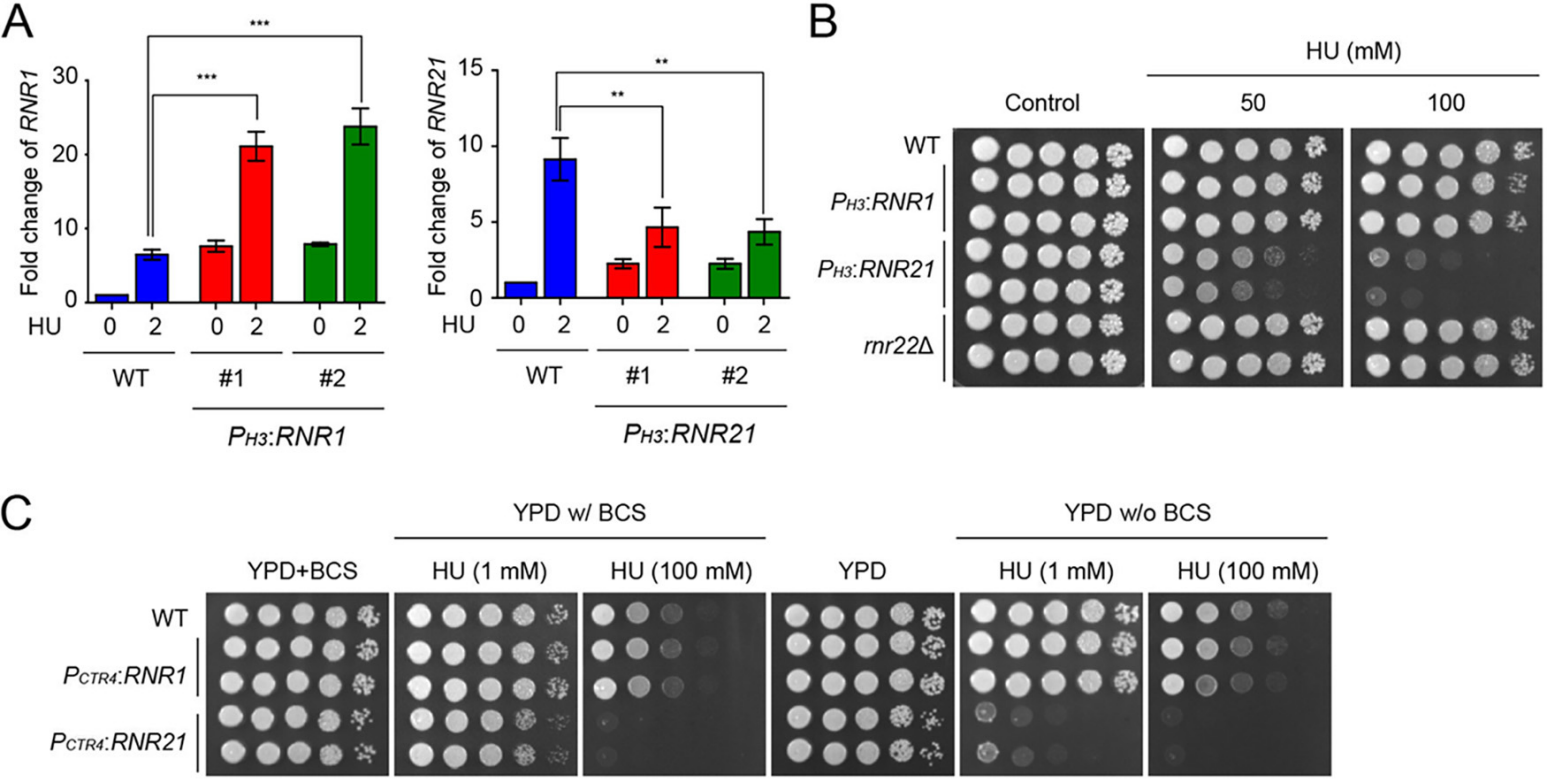
Transcriptional changes in *RNR21* resulted in increased sensitivity in response to DNA replication stress. (A) Expression of *RNR1* and *RNR21* in *P_H3_*:*RNR1* and *P_H3_*:*RNR21* strains in response to HU. qRT-PCR analysis was performed using cDNA synthesized from total RNA isolated from H99, *P_H3_*:*RNR1*, and *P_H3_*:*RNR21* strains treated with 50 mM HU. (B) The *P_H3_*:*RNR21* strains were highly susceptible to HU. Strains were cultured in liquid YPD medium at 30°C for 16 h. The serially diluted cells were spotted onto the solid media containing the indicated concentration of HU. (C) The WT, *P_CTR4_*:*RNR1*, and *P_CTR4_*:*RNR21* strains were cultured in liquid YPD medium at 30°C for 16 h. Next, the strains were 10-fold serially diluted and spotted on the YPD medium containing the indicated concentration of HU in the presence or absence of BCS. The cells were further incubated at 30°C for 3 days. Statistical significance of differences was determined by analysis of variance (ANOVA) with Bonferroni’s test (Prizm). Error bars indicate standard error of the mean (***, *P < *0.001 and **, *P < *0.01).

Our experimental results showed that *RNR1* and *RNR21* expression was induced in response to DNA replication stress (treatment with HU) and DNA damage stress (treatment with MMS or gamma radiation) and *RNR22* expression was only induced in response to gamma radiation, indicating that RNRs might be involved in DNA replication and DNA damage stresses. To investigate this, we first constructed *RNR22* deletion strains and constitutive *RNR1-* or *RNR21-*overexpression strains by replacing its native promoter with the histone 3 (H3) promoter, because *RNR1* and *RNR21* are required for viability. We found that *H3* promoter replacement increased basal *RNR1* expression levels, which were almost equivalent to HU-induced *RNR1* expression levels (Fig. 5A). Interestingly, however, HU treatment further increased the expression of *RNR1* in the P*_H3_:RNR1* strain (Fig. 5A), implying that an enhancer outside the replaced *RNR1* promoter region or unknown factors may act on HU-mediated induction because *H3* promoter, *per se*, was not induced under DNA replication stress (Data not shown). In contrast, *H3* promoter replacement increased basal *RNR21* expression levels, but *RNR21* induction in *P_H3_:RNR21* strains was much lower than that in WT in the presence of HU (Fig. 5A). Next, we performed a survival test in response to DNA replication and DNA damage stress, using these strains. Notably, strains containing *P_H3_*:*RNR21* showed significant growth defects in response to HU, whereas the *P_H3_*:*RNR1* strain and *rnr22*Δ mutants exhibited the WT level of resistance to HU (Fig. 5B). In the case of DNA damage stress, *P_H3_*:*RNR1*, *P_H3_*:*RNR21,* and *rnr22*Δ mutant strains were as resistant to DNA damage stress as WT (Fig. S3).

Next, we performed phenotypic analyses using the *P_CTR4_*:*RNR1* and *P_CTR4_*:*RNR21* strains to further demonstrate the roles of *RNR1* and *RNR21* in DNA damage and replication stress. Consistent with the phenotype of the strains containing *P_H3_*:*RNR1*, *P_CTR4_*:*RNR1* strains were as resistant to HU as WT, regardless of the presence of BCS. Notably, the *P_CTR4_*:*RNR21* strains showed significant growth defects in response to HU (Fig. 5C). Furthermore, the *P_CTR4_*:*RNR21* strains were slightly more resistant to HU in the presence of BCS than in the absence of BCS. Under DNA damage stress, *P_CTR4_*:*RNR21* strains, but not *P_CTR4_*:*RNR1* strains, showed growth defects in response to MMS and 4-NQO in the presence and absence of BCS (Fig. S3). Under BCS treatment, the growth inhibition of *P_CTR4_*:*RNR21* strains was partially rescued in response to MMS and radiation exposure compared with that in the absence of BCS. However, the *P_CTR4_*:*RNR1* strains showed resistance similar to that of WT to MMS, 4-NQO and radiation exposure (Fig. S3). The growth of the WT and tested strains was slightly more retarded in the presence of both BCS and stress-inducing agents than in the presence of the stress-inducing agents alone, probably due to reduced intracellular Cu^2+^ levels resulting from the Cu^2+^-chelating activity of BCS (Fig. 5C; Fig. S3). This phenomenon has also been observed in previous studies (28, 29). Taken together, transcriptional changes in *RNR21* may contribute to DNA replication and DNA damage stress in C. neoformans.

## DISCUSSION

Given that RNR is involved in the rate-limiting step for providing the dNTPs required for DNA synthesis and the DNA repair process, most genes encoding them are essential for viability. In S. cerevisiae, *RNR1*, *RNR2*, and *RNR4*, but not *RNR3*, are essential for viability. The fission yeast Schizosaccharomyces pombe contains *CDC22* and *SUC22*, encoding the large and small subunits of RNR, respectively, and these genes are also essential for viability (30). Furthermore, C. albicans *RNR1* and *RNR21* are essential genes, whereas *RNR3* and *RNR22* are predicted to be nonessential (31, 32). Similar to other yeasts, C. neoformans *RNR1* and *RNR21* are also required for survival. Notably, nonessential *RNR* paralogous genes for each large and small subunit seemed to compensate for the loss of the counterpart gene. In S. cerevisiae, *RNR3* overexpression suppresses the decreased viability caused by reduced levels of *RNR1*, whereas *RNR2* and *RNR4*, which are essential genes, do not compensate for the viability of each other (5). In contrast, our present study revealed that *RNR22* overexpression compensated for the reduced viability caused by *RNR21* suppression. ScRnr4 lacks several conserved amino acids required for iron-binding and cannot form canonical tyrosyl radicals (33) whereas CnRnr22 contains conserved amino acids. This conserved motif of Rnr22 may compensate for the loss of Rnr21. In contrast to yeasts, information on the role of viability of RNR genes in filamentous fungi is limited. Neurospora crassa genome contains genes encoding a large and small subunit of RNR and the gene (UN-24) encoding a large subunit of RNR is also critical for viability, whereas the function of the gene (NCU07887) encoding a small subunit of RNR has not been characterized yet (34). Likewise, the roles of *rnsA* and *rnrA*, encoding a large and small subunit of RNR, respectively, in viability have not been determined in A. nidulans. Therefore, the roles of RNRs in the viability of filamentous fungi require further characterization.

Although the expression levels of most *RNR* genes are altered in response to DNA damage stress and are evolutionarily conserved from prokaryotes to mammals, their expression patterns are divergent in species and are induced in a DNA damage stress-dependent manner. In mammals, the expression of R1, encoding a large subunit of RNR, and p53R2, encoding a small subunit of RNR, is induced under DNA damage stress (35, 36). However, the expression of R2, which encodes a small subunit of RNRs, is constant or decreases depending on the DNA damage insults (35, 37). Similar to mammals, in Escherichia coli, the expression levels of *nrdA* and *nrdB*, encoding the large and small subunits of RNR, respectively, are also dependent on DNA damage stress. Upon UV exposure, *nrdA* and *nrdB* expression increases (38). However, *nrdA* expression is also induced in the presence of bleomycin and mitomycin C, whereas *nrdB* expression does not change under these conditions (39). Recently, Cohen et al. reported that *RNR1* and *RNR2* expression does not change in response to DNA damage insults such as HU and MMS at the transcriptional and translational levels in Fusarium oxysporum (40). However, *RNR1* expression increases in response to HU, whereas *RNR2* expression does not change upon exposure to HU and MMS in Fusarium *verticillioides* (40). Consistent with previous results, *RNR1* and *RNR21* expressions were highly increased following treatment with HU, 4-NQO, MMS, and gamma radiation, whereas *RNR22* expression was induced to a lesser extent in C. neoformans in response to gamma radiation and MMS. Taken together, RNR genes are induced in response to DNA damage stress in a damage type- and species-dependent manner.

**FIG 6:**
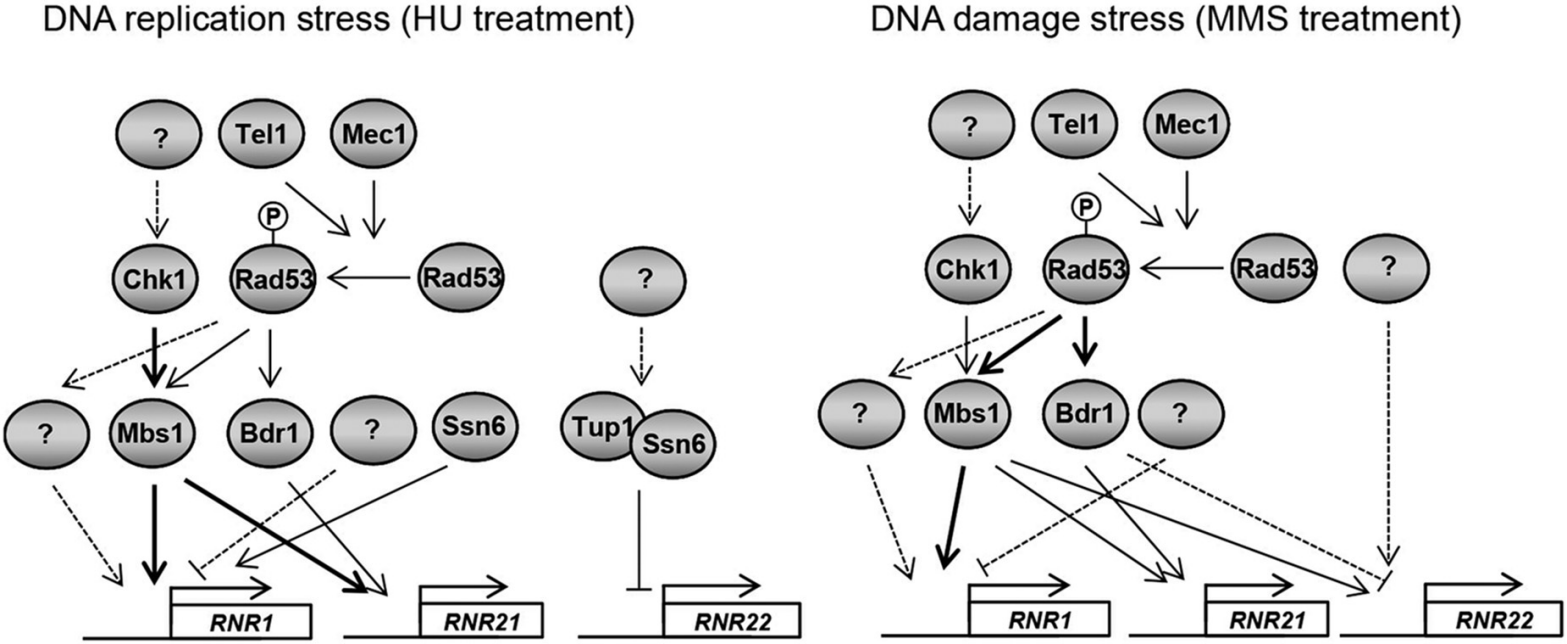
Proposed model of Rad53- and Chk1-dependent DNA replication and damage stresses. In response to HU treatment (DNA replication stress), Chk1, rather than Rad53, regulates *RNR1* expression through the Mbs1 transcription factor. In contrast, Chk1 and Rad53 cooperatively control expression levels of *RNR21* through Mbs1 and Bdr1 transcription factors. The Ssn6-Tup1 complex suppresses *RNR22* expression. In response to MMS treatment (DNA damage stress), Chk1 and Rad53 equally contribute to *RNR1* induction, whereas Rad53 and Chk1 play major and minor roles, respectively, in *RNR21* induction. Chk1 and Rad53 are not involved in the regulation of *RNR22* under both DNA replication and damage stress. Mbs1 plays a major role in MMS-mediated induction of *RNR1*, *RNR21*, and *RNR22*, whereas Bdr1 is involved in *RNR21* and *RNR22* induction in an opposite manner.

Although the C. neoformans DNA repair pathway composed of Rad53 and Chk1 is mainly critical for the induction of *RNR* genes, similar to the S. cerevisiae Mec1-Rad53-Dun9 pathway, there is evidence that the regulatory mechanisms of RNRs in C. neoformans are divergent compared with those in S. cerevisiae. First, Rad53 and Chk1 cooperatively or independently regulate the expression of *RNR1* and *RNR21* depending on the DNA replication and damage stresses in C. neoformans (Fig. 6). Under DNA replication stress induced by HU treatment, Chk1, rather than Rad53, mainly controlled the expression of *RNR1*, whereas Chk1 and Rad53 cooperatively regulated *RNR1* expression under DNA damage stress caused by MMS treatment or gamma radiation exposure. In contrast to *RNR1* expression, Rad53, rather than Chk1, was a major factor responsible for the regulation of *RNR21* during DNA damage stress. Second, the orthologs of *CRT1*, *IXR1*, *SML1*, and *DIF1* required to regulate RNRs in S. cerevisiae have not been identified in the Cryptococcus genome. Instead, Mbs1 plays a more critical role in controlling *RNR1* and *RNR21* expression than Bdr1. Notably, *RNR1* expression in the *mbs1*Δ mutant was intrinsically higher than that in the WT, indicating that another factor (or other factors) may compensate for the loss of *MBS1* at the basal level. Third, the expression patterns of RNRs controlled by the Ssn6-Tup1 complex differ from those in S. cerevisiae. The Ssn6-Tup1 complex with Crt1 suppresses the induction of *RNR2*, *RNR3*, and *RNR4* expression in the absence of DNA damage stress (6, 27). However, the Ssn6-Tup1 complex in C. neoformans negatively regulated the expression of *RNR22*, but not that of *RNR1* and *RNR21*. Given that Ssn6-Tup1 *per se* does not contain a DNA-binding domain, a novel transcription factor may interact with Ssn6-Tup1 during DNA replication stress.

Although both *RNR1* and *RNR21* expression is regulated by the Rad53-Chk1-Bdr1 pathway and Mbs1 in response to environmental cues, the effect of its expression level *per se* on its function is divergent. In S. cerevisiae, mutation of the Rnr1 allosteric sites results in high levels of dNTP production, which renders strains resistant to DNA damage stress and leads to a high frequency of mutation rates (41, 42). Notably, the effect of *RNR1* overexpression varies depending on the genetic background. *RNR1* overexpression increases resistance to DNA damage in the WT strain and suppresses lethality in *rad53*Δ and *mec1*Δ mutants (26, 43). However, *RNR1* overexpression in strains lacking the gene encoding the subunit of the replication origin recognition complex decreases cell viability (26). In C. neoformans, *RNR1* overexpression in WT did not increase DNA damage resistance. However, *RNR1* overexpression in the *mbs1*Δ mutant increased DNA damage sensitivity. This might be due to genetic instability caused by high mutation rates or other reasons. At this point, we need to further elucidate the mechanism by which *RNR1* overexpression affects the DNA damage response in the diverse genetic backgrounds of C. neoformans. In C. neoformans, the change in *RNR21* expression is more critical for its role than *RNR1*. First, the growth of the *P_CTR4_*:*RNR21* strains were significantly reduced compared with that of the *P_CTR4:_RNR1* strains under promoter-repressed conditions. Furthermore, *P_CTR4_*:*RNR21* strains exhibited sensitivity to DNA damage and DNA replication stress. Second, the *P_H3_*:*RNR21* strains were more susceptible to HU treatment than *P_H3_*:*RNR1* strains. Given that HU inhibits the radical reaction in a small subunit of the Rnr complex, reduced *RNR21* induction in the *P_H3_*:*RNR21* strain would result in significant susceptibility to HU treatment. Taken together, transcriptional regulation is important for the role of Rnr21 during DNA replication and DNA damage stress.

## MATERIALS AND METHODS

### Strains, growth conditions, and stress-resistance tests.

The C. neoformans strains used in the present study are listed in Table S1. The strains were cultured on yeast extract peptone dextrose (YPD) medium for the stress resistance test. Each strain was incubated for 16 h at 30°C in the liquid YPD medium. Next, the cells were serially diluted (1 to 10^4^ dilutions) and spotted onto a solid YPD medium containing the indicated concentration of HU and DNA damage insults. For the viability tests, the strains were cultured for 16 h at 30°C in liquid YPD medium. Cells were serially (1 to 10^4^ dilutions) and spotted onto a solid yeast nitrogen base (YNB) or YPD medium containing the indicated concentration of CuSO_4_ or BCS, a copper chelator. The cells were further incubated at 30°C for 1 to 3 days and photographed daily.

### Construction of strains with the *CTR4* promoter or *H3* promoter replacement of *RNR1*, *RNR21*, and *RNR22*.

To replace each native gene promoter with a copper-regulated *CTR4* promoter, we constructed an RNR promoter replacement cassette as follows. Primer pairs CTR4-L1/L2 and CTR4-R1/R2 were used to amplify the 3′-flanking region of the RNR promoter and the 5′-flanking region of the RNR exon, respectively. The *NAT-CTR4* promoter was amplified using primers B354 and B355 using pNAT-CTR4 as a template. The RNR promoter replacement cassette was produced by double-joint PCR (DJ-PCR) using the primer pairs CTR4-L1/B1555 and CTR4-R2/B1554. The two PCR products were mixed and biolistically transformed into C. neoformans H99. Stable transformants selected on YPD medium containing nourseothricin were screened for correct insertion by diagnostic PCR. Finally, Southern blot analysis was performed to determine the correct genotype with promoter replacement strains (44).

To replace each native *RNR22* promoter with a histone H3 promoter, we generated an *RNR22* promoter replacement cassette as follows. In the first round of PCR, the J1583/J1611 and J1655/J1642 primer pairs were used to amplify the 5′-flanking and 5′-coding regions, respectively. The NEO-H3 promoter region was amplified using the B4017/B4018 primer pair, with pNEO-H3 as a template. In the second-round of PCR, the J1583/B1887 and J1642/B1886 primer pairs were used to amplify the 5′- and 3′-regions of the *P_H3_*:*RNR22* replacement cassettes, respectively. The NEO-marked H3 promoter was introduced into the native promoter region of *RNR22* in the *P_CTR4_*:*RNR21* strain (KW1418). Stable transformants on YPD medium containing G418 (100 μg/mL) were screened using diagnostic PCR. Next, the correct genotype of positive transformants was confirmed by Southern blotting analysis as previously described (44). Constitutive overexpression of *RNR22* was verified by qRT-PCR using *RNR22* gene-specific primers (J122/J123).

To construct the *P_H3_*:*RNR1* or *P_H3_*:*RNR21* strains, we replaced the native promoter of its gene with the *H3* promoter. The cassettes for *P_H3_*:*RNR1-NEO* and *P_H3_*:*RNR21-NEO* were generated as follows. In the first round of PCR, primer pairs OE-L1/OE-L2 and OE-R1/OE-R2 were used to amplify the 5′-flanking region and 5′-coding regions, respectively. The NEO-H3 promoter region was amplified using primers B4017/B4018 with pNEO-H3 as a template. In the second round of PCR, the primer pairs OE-L1/B1887 and OE-R2/B1886 were used to amplify the 5′- and 3′-regions of *P_H3_:RNR1* or *P_H3_:RNR21* replacement cassettes. *NEO*-marked replacement cassettes were then introduced into the H99 strain. Next, stable transformants on YPD medium containing G418 were screened by diagnostic PCR and the correct genotype of these positive strains was verified by Southern blotting analysis. The expression levels of *RNR1* and *RNR21* were confirmed by qRT-PCR using *RNR1* or *RNR21* gene-specific primers (*RNR1*: J118/J119 and *RNR21*: J120/J121) in the presence of HU.

### Construction of *the rnr22*Δ, *ssn6*Δ, *tup1*Δ, and *bdr1*Δ *mbs1*Δ double mutants.

To disrupt *SSN6*, *TUP1*, and *RNR22* in C. neoformans, we obtained information regarding the genomic structure and sequences of these genes from FungiDB (www.fungidb.org). To construct the *ssn6*Δ, *tup1*Δ, and *rnr22*Δ mutants, *SSN6*, *TUP1*, and *RNR22* gene disruption cassettes were generated by double-joint PCR (DJ-PCR) as follows. Primer pairs L1/L2 and R1/R2 were used to amplify the 5′- and 3′-flanking regions of each gene. The M13Fe and M13Re primers were used to amplify the Nat^r^ dominant selectable marker. Each gene disruption cassette was generated by DJ-PCR, as previously described (44, 45). Each gene disruption cassette was biolistically inserted into C. neoformans H99. Stable transformants were selected on YPD medium containing nourseothricin and screened by diagnostic PCR. To generate *bdr1*Δ *mbs1*Δ double mutants, *BDR1* gene disruption cassettes with a Neo^r^-dominant selectable marker were generated by DJ-PCR. The *BDR1* disruption cassette was biolistically transformed into the *mbs1*Δ mutant. Stable transformants were selected on YPD medium containing neomycin and screened by diagnostic PCR.

Southern blot analysis was performed to verify the correct genotype of all mutants with gene-specific probes (44). All primer information for the disruption of *SSN6*, *TUP1*, *RNR22*, and *BDR1* are listed in Table S2.

### Total RNA isolation, cDNA synthesis, and qRT-PCR.

To measure the expression levels of RNR genes under HU and MMS treatment, total RNA was isolated from the WT, *rad53*Δ, *chk1*Δ, *rad53*Δ *chk1*Δ double mutant, *bdr1*Δ, *mbs1*Δ, *ssn6*Δ, *tup1*Δ, and *bdr1*Δ *mbs1*Δ double mutants. The strains were cultured in 20 mL of liquid YPD medium for 16 h at 30°C. The grown cells were inoculated into 100-mL fresh YPD medium and adjusted to an OD_600_ of 0.2. The strains were further incubated at 30°C until the OD_600_ of the culture medium reached approximately 0.6 to 0.7. The cells (50 mL) were pelleted by centrifugation for the zero-time (basal) sample and the remaining cells were treated with the indicated concentration of HU (final concentration: 50 mM) or MMS (final concentration: 0.02%). After 1 h, the cells were pelleted by centrifugation and stored in liquid nitrogen before total RNA isolation. For gamma radiation exposure, 50 mL of the 150 mL culture was used as the basal sample and the remaining 100-mL culture was exposed to radiation for 1 h. After radiation exposure, a 50-mL culture was sampled at 30 and 60 min during incubation. All samples were lyophilized overnight and total RNA was extracted from the dried cells using TRIzol reagent (EasyBlue; intron), as previously described (46). Total RNA was further purified using an RNeasy spin column (Qiagen) according to the manufacturer’s instructions. cDNA was synthesized with the PrimeScript 1st strand cDNA Synthesis Kit (TaKaRa) using purified RNA as a template. To measure the expression levels of target genes, we performed qRT-PCR analysis using the gene-specific primers listed in Table S2 and the CFX96 real-time PCR detection system (Bio-Rad). The relative expression of the target genes was determined using the 2^-ΔΔCt^ method (47) and statistical analyses were performed using one-way analysis of variance (ANOVA) with Bonferroni’s multiple-comparison test (GraphPad Software Inc.).
