# Supplementary material for: Essential Roles of Ribonucleotide Reductases under DNA Damage and Replication Stresses in Cryptococcus neoformans
Source: Microbiol Spectr. 2022 Jun 23;10(4):e01044-22. doi: 10.1128/spectrum.01044-22 (PMC9431586; doi:10.1128/spectrum.01044-22)
Supplement: Supplemental file 1 — Supplemental material. Download spectrum.01044-22-s0001.pdf, PDF file, 0.6 MB [file spectrum.01044-22-s0001.pdf]

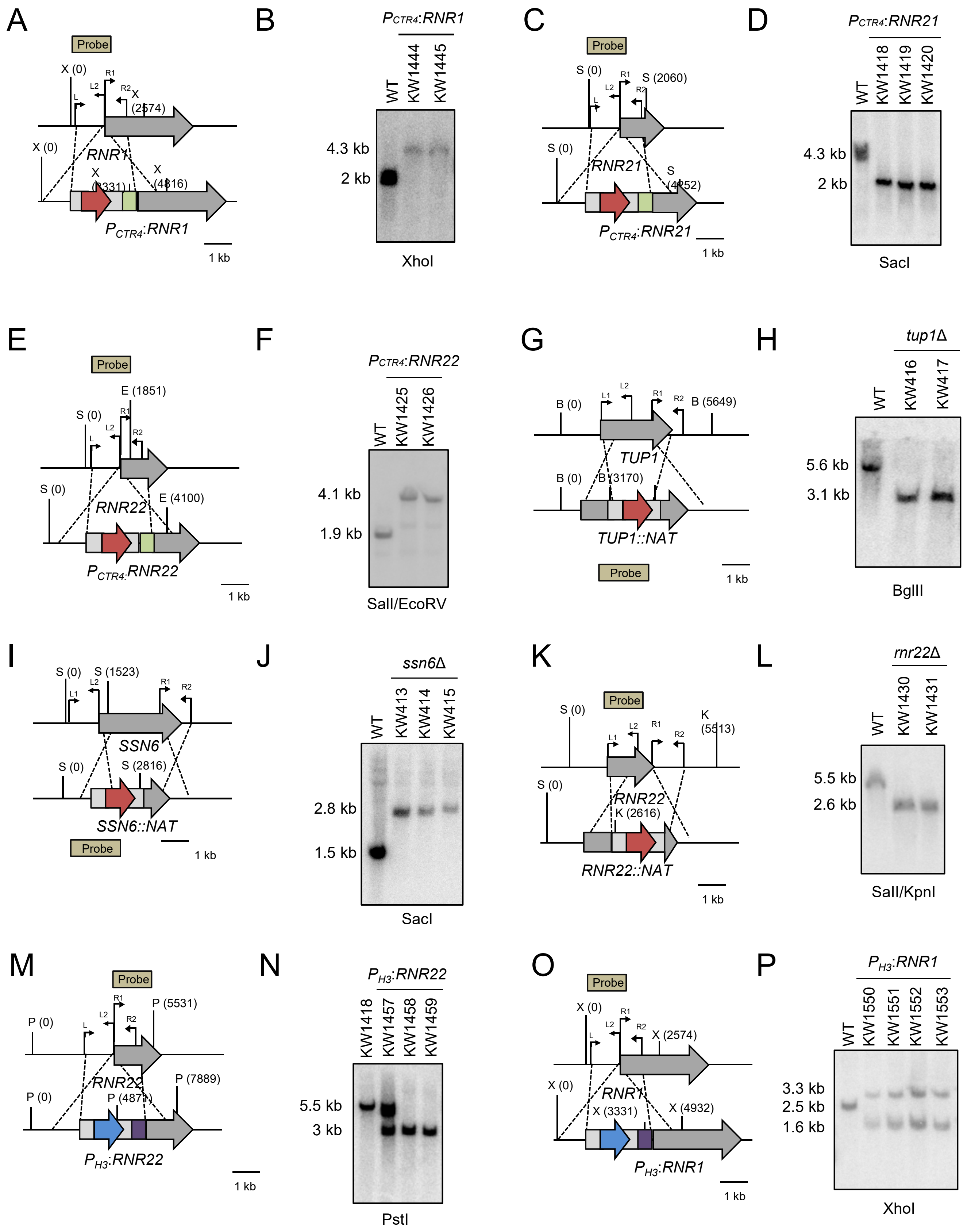
A
Probe
X (0)
X (2574)
RNR1
X (3331)
X (4816)
P_CTR4:RNR1
1 kb
B
P_CTR4:RNR1
WT
KW1444
KW1445
4.3 kb
2 kb
XhoI
C
Probe
S (0)
S (2060)
RNR21
S (4252)
P_CTR4:RNR21
1 kb
D
P_CTR4:RNR21
WT
KW1418
KW1419
KW1420
4.3 kb
2 kb
SacI
E
Probe
E (1851)
S (0)
RNR22
E (4100)
P_CTR4:RNR22
1 kb
F
P_CTR4:RNR22
WT
KW1425
KW1426
4.1 kb
1.9 kb
SalI/EcoRV
G
B (0)
B (5649)
TUP1
B (3170)
TUP1::NAT
Probe
1 kb
H
tup1Δ
WT
KW416
KW417
5.6 kb
3.1 kb
BglII
I
S (0)
S (1523)
SSN6
S (2816)
SSN6::NAT
Probe
1 kb
J
ssn6Δ
WT
KW413
KW414
KW415
2.8 kb
1.5 kb
SacI
K
Probe
S (0)
K (5513)
RNR22
K (2616)
RNR22::NAT
1 kb
L
rnr22Δ
WT
KW1430
KW1431
5.5 kb
2.6 kb
SalI/KpnI
M
Probe
P (0)
P (5531)
RNR22
P (7889)
P (4871)
P_H3:RNR22
1 kb
N
P_H3:RNR22
KW1418
KW1457
KW1458
KW1459
5.5 kb
3 kb
PstI
O
Probe
X (0)
X (2574)
RNR1
X (3331)
X (4932)
P_H3:RNR1
1 kb
P
P_H3:RNR1
WT
KW1550
KW1551
KW1552
KW1553
3.3 kb
2.5 kb
1.6 kb
XhoI

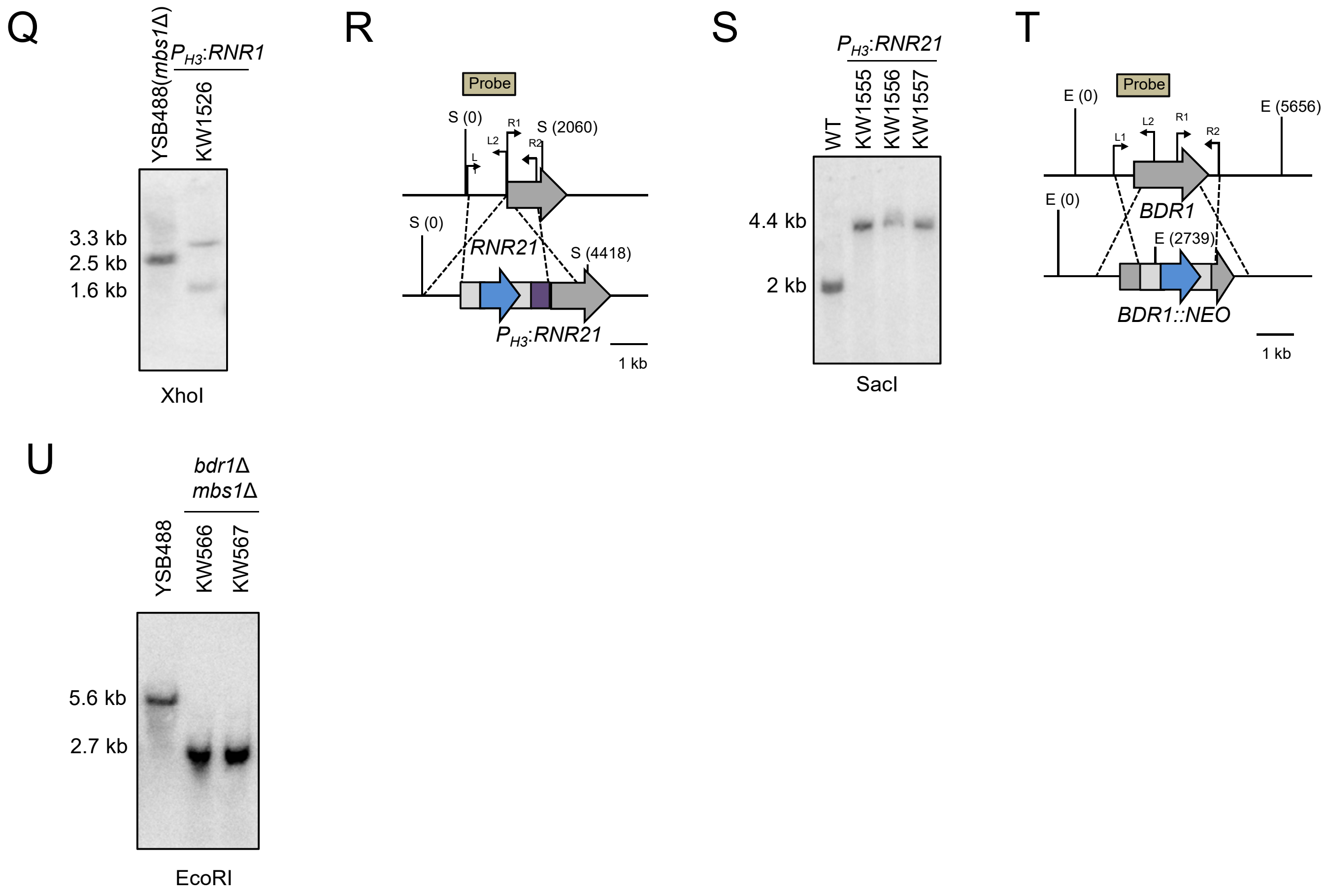

Figure S1. Construction of *ssn6*Δ, *tup1*Δ, *rnr22*Δ, *bdr1*Δ *mbs1*Δ double mutant, P_{CTR4}:*RNR1*, P_{CTR4}:*RNR21*, P_{CTR4}:*RNR22*, P_{H3}:*RNR1*, P_{H3}:*RNR21*, and P_{H3}:*RNR22* strains. (A, C, E, G, I, K, M, O, R and T) Diagram for deletions of *SSN6*, *TUP1*, and *RNR21* with *NAT* marker, *BDR1* with *NEO* marker, and replacement of native promoter of *RNR1*, *RNR21,* and *RNR22* with H3 promoter. (B, D, F, H, J, L, N, P, Q, S, and U) Verification of correct genotypes of the strains. The membrane was hybridized with the corresponding gene-specific probe, washed, and developed.

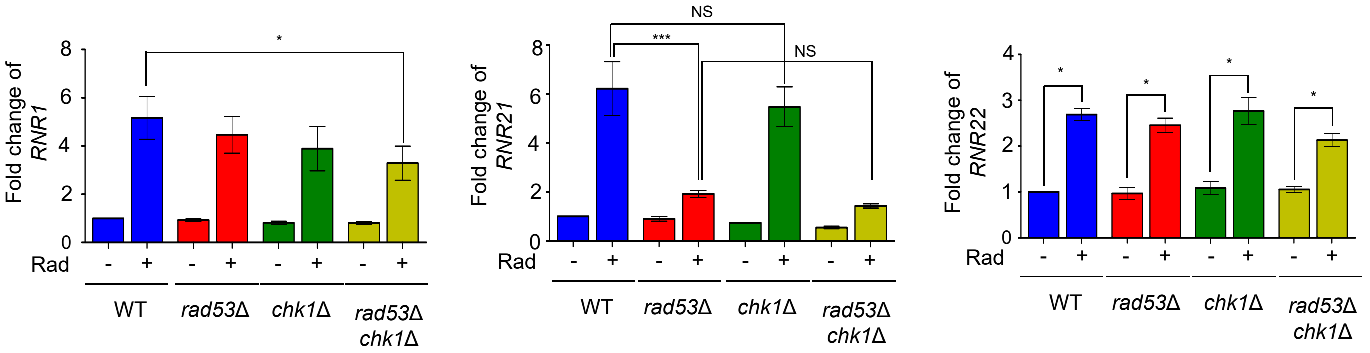

Figure S2 Expression levels of *RNR1*, *RNR21* and *RNR22* in WT, *rad53*Δ, *chk1*Δ and *rad53*Δ *chk1*Δ double mutant after γ-radiation exposure. The qRT-PCR analysis was performed using cDNA synthesized from total RNA isolated from WT H99, *rad53*Δ, *chk1*Δ, and *rad53*Δ *chk1*Δ double mutant. Three independent biological samples were analysed with duplicate technical replicates. Error bars indicate standard error of the mean (S. E. M). (* $p < 0.05$, *** $p < 0.001$ and NS: non-significant). **.**

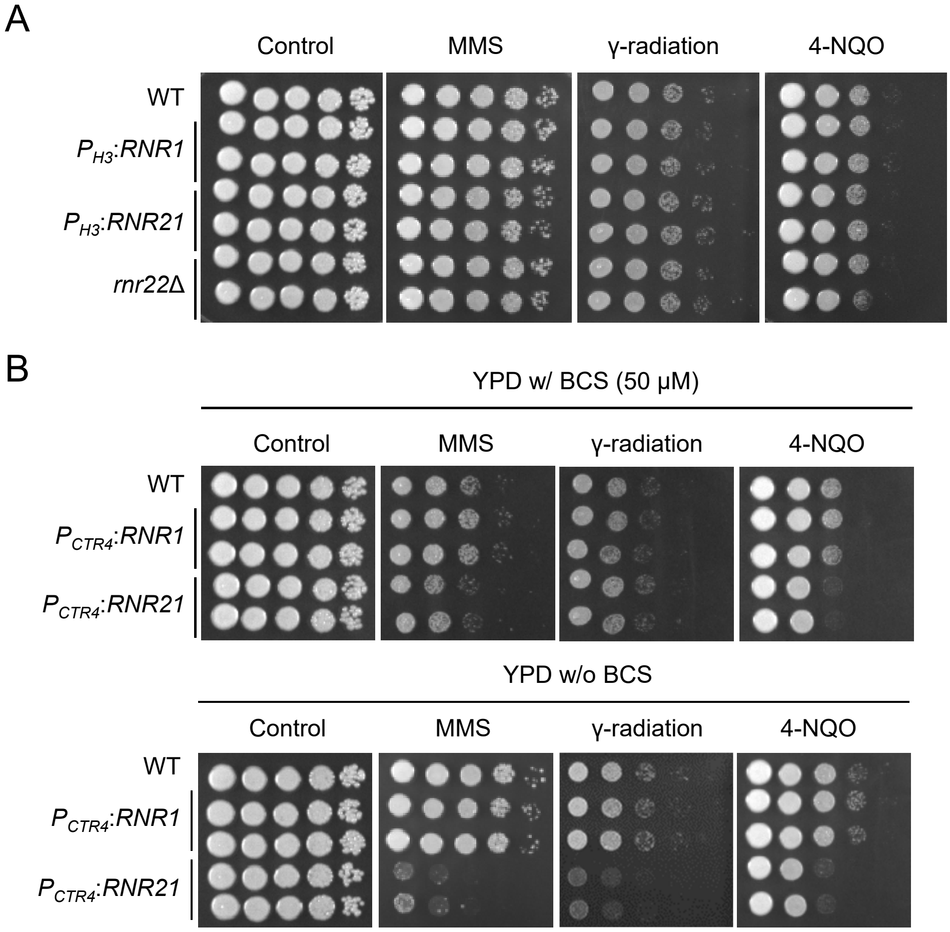

Figure S3. Phenotypic analyses of *rnr22*Δ, P_{H3}:*RNR1*, P_{H3}:*RNR21,* P_{CTR4}:*RNR1*, and P_{CTR4}:*RNR21* strains under genotoxic stress. (A and B) Strains were cultured in liquid yeast extract peptone dextrose (YPD) medium for 16 h and serially diluted cells were spotted onto the YPD media containing the genotoxic stress reagents. For radiation-resistance assay, the serially diluted cells were spotted onto the YPD media and then exposed to the radiation. Strains were further incubated at 30°C for 1–3 days.

Table S1. Strains used in this study

Strain	Genotype	Parent	Reference
H99	*MATα*		(1)
KW137	*MATα CNAG_02589* (*BDR1*)::*NAT #230*	H99	(2)
KW191	*MATα CNAG_03167* (*CHK1*)::*NAT#234*	H99	(3)
KW250	*MATα CNAG_05216* (*RAD53*)::*NAT#184 CNAG_03167* (*CHK1*)::*NEO*	YSB3785	(3)
YSB3785	*MATα CNAG_05216* (*RAD53*)::*NAT#184*	H99	(4)
YSB488	*MATα CNAG_07464* (*MBS1*)::*NAT #150*	H99	(5)
KW566	*MATα CNAG_07464* (*MBS1*)::*NAT #150 CNAG_02589* (*BDR1*)::*NEO*	YSB488	This study
KW567	*MATα CNAG_07464* (*MBS1*)::*NAT #150 CNAG_02589* (*BDR1*)::*NEO*	YSB488	This study
KW413	*MATα CNAG_01902* (*SSN6*)::*NAT #56*	H99	This study
KW414	*MATα CNAG_01902* (*SSN6*)::*NAT #56*	H99	This study
KW415	*MATα CNAG_01902* (*SSN6*)::*NAT #56*	H99	This study
KW416	*MATα CNAG_02153* (*TUP1*)::*NAT #56*	H99	This study
KW417	*MATα CNAG_02153* (*TUP1*)::*NAT #56*	H99	This study
KW1430	*MATα CNAG_02663* (*RNR22*)::*NAT #56*	H99	This study
KW1431	*MATα CNAG_02663* (*RNR22*)::*NAT #56*	H99	This study
KW1444	*MATα* P_{CTR4}:*CNAG_02208* (*RNR1*)-*NAT*	H99	This study
KW1445	*MATα* P_{CTR4}:*CNAG_02208* (*RNR1*)-*NAT*	H99	This study
KW1418	*MATα* P_{CTR4}:*CNAG_01915* (*RNR21*)-*NAT*	H99	This study
KW1419	*MATα* P_{CTR4}:*CNAG_01915* (*RNR21*)-*NAT*	H99	This study
KW1420	*MATα* P_{CTR4}:*CNAG_01915* (*RNR22*)-*NAT*	H99	This study
KW1421	*MATα* P_{CTR4}:*CNAG_01915* (*RNR22*)-*NAT*	H99	This study
KW1425	*MATα* P_{CTR4}:*CNAG_02663* (*RNR22*)-*NAT*	H99	This study
KW1426	*MATα* P_{CTR4}:*CNAG_02663* (*RNR22*)-*NAT*	H99	This study
KW1550	*MATα* P_{H3}:*CNAG_02208* (*RNR1*)-*NEO*	H99	This study
KW1551	*MATα* P_{H3}:*CNAG_02208* (*RNR1*)-*NEO*	H99	This study
KW1552	*MATα* P_{H3}:*CNAG_02208* (*RNR1*)-*NEO*	H99	This study
KW1553	*MATα* P_{H3}:*CNAG_02208* (*RNR1*)-*NEO*	H99	This study
KW1526	*MATα* P_{H3}:*CNAG_02208* (*RNR1*)-*NEO CNAG_07464* (*MBS1*)::*NAT #150*	YSB488	This study
KW1555	*MATα* P_{H3}:*CNAG_01915* (*RNR21*)-*NEO*	H99	This study
KW1556	*MATα* P_{H3}:*CNAG_01915* (*RNR21*)-*NEO*	H99	This study
KW1557	*MATα* P_{H3}:*CNAG_01915* (*RNR21*)-*NEO*	H99	This study
KW1458	*MATα* P_{CTR4}:*CNAG_01915* (*RNR21*)-*NAT* P_{H3}:*CNAG_02663* (*RNR22*)-*NEO*	KW1418	This study
KW1459	*MATα* P_{CTR4}:*CNAG_01915* (*RNR21*)-*NAT* P_{H3}:*CNAG_02663* (*RNR22*)-*NEO*	KW1418	This study

Each *NAT-STM#* indicates the Nat^r marker with a unique signature tag.

REFERENCES

1. Perfect JR, Ketabchi N, Cox GM, Ingram CW, Beiser CL. 1993. Karyotyping of *Cryptococcus neoformans* as an epidemiological tool. J Clin Microbiol 31:3305-9.
2. Jung KW, Yang DH, Kim MK, Seo HS, Lim S, Bahn YS. 2016. Unraveling Fungal Radiation Resistance Regulatory Networks through the Genome-Wide Transcriptome and Genetic Analyses of *Cryptococcus neoformans*. mBio 7.
3. Jung KW, Lee Y, Huh EY, Lee SC, Lim S, Bahn YS. 2019. Rad53- and Chk1-Dependent DNA Damage Response Pathways Cooperatively Promote Fungal Pathogenesis and Modulate Antifungal Drug Susceptibility. mBio 10.
4. Lee KT, So YS, Yang DH, Jung KW, Choi J, Lee DG, Kwon H, Jang J, Wang LL, Cha S, Meyers GL, Jeong E, Jin JH, Lee Y, Hong J, Bang S, Ji JH, Park G, Byun HJ, Park S, Park YM, Adedoyin G, Kim T, Averette AK, Choi JS, Heitman J, Cheong E, Lee YH, Bahn YS. 2016. Systematic fungal analysis of kinases in the fungal pathogen *Cryptococcus neoformans*. Nat Commun 7:12766.
5. Song MH, Lee JW, Kim MS, Yoon JK, White TC, Floyd A, Heitman J, Strain AK, Nielsen JN, Nielsen K, Bahn YS. 2012. A flucytosine-responsive Mbp1/Swi4-like protein, Mbs1, plays pleiotropic roles in antifungal drug resistance, stress response, and virulence of *Cryptococcus neoformans*. Eukaryot Cell 11:53-67.

Table S2. Primers used in this study

Primer Name	Sequence (5'—3')	Comment
B79	TGTGGATGCTGGCGGAGGATA	Screening primer on *ACT1* promter
B1026	GTAAAACGACGGCCAGTGAGC	M13 forward (extended)
B1027	CAGGAAACAGCTATGACCATG	M13 reverse (extended)
B1454	AAGGTGTTCCCCGACGACGAATCG	NSL2
B1455	AACTCCGTCGCGAGCCCCATCAAC	NSR2
B1886	TGGAAGAGATGGATGTGC	NSL-NEO
B1887	ATTGTCTGTTGTGCCCAG	NSR-NEO
B354	GCATGCAGGATTCGAGTG	Primer 1 for *CTR4* promoter cassette
B355	GATTGGTGAAGTCGTTGTCG	Primer 2 for *CTR4* promoter cassette
B354	GCATGCAGGATTCGAGTG	Primer 1 for *H3* overexpression cassette
B4018	GTGATAGATGTGTTGTGGTG	Primer 2 for *H3* overexpression cassette
B679	CGCCCTTGCTCCTTCTTCTATG	*ACT1* qRT primer 1
B680	GACTCGTCGTATTCGCTCTTCG	*ACT1* qRT primer 2
J118	CAGAAGGCTGTCATCGACC	*RNR1* (CNAG_02208) qRT primer 1
J119	AGTGAGCTGAGGGAACGAG	*RNR1* (CNAG_02208) qRT primer 2
J120	ATTAAGGACACTGCTCGACG	*RNR21* (CNAG_01915) qRT primer 1
J121	TGGTCAGAGATCCATCGAAG	*RNR21* (CNAG_01915) qRT primer 2
J122	TACCGATGCTCTTCCTTGTG	*RNR21* (CNAG_02663) qRT primer 1
J123	ATAGCCCAAGTCAACGACG	*RNR22* (CNAG_02663) qRT primer 2
J668	CACACCGAGCCCCGTCGCGA	*MBS1* (CNAG_07464) qRT primer 1
J669	ATTTTGGGACAGCGTCAC	*MBS1* (CNAG_07464) qRT primer 2
J1570	TGCACGATTTCGTCTGCTG	*RNR1* (CNAG_02208) –5' screening primer for CTR4
J1571	ACCCGATAACACAGGACAC	*RNR1* (CNAG_02208) – left flanking primer 1 for CTR4
J1635	GCCACTCGAATCCTGCATGCGAATCGTGGAAGAAGAGGGA	*RNR1* (CNAG_02208) – left flanking primer 2 for CTR4
J1636	CGACAACGACTTCACCAATCTCCCTCTTCTTCCACGATTC	*RNR1* (CNAG_02208) – right flanking primer 1 for CTR4
J1574	CCACGAGGAAGCATGATGAC	*RNR1* (CNAG_02208) – right flanking primer 2 for CTR4
J1575	TTCGGGCAGCAAGGATAG	*RNR1* (CNAG_02208) – probe primer for Southern blot
J1576	TCGTCATCGTCCATTTCCTC	*RNR21* (CNAG_01915) –5' screening primer for CTR4
J1577	TGGAAGACTTTGCCTGGAC	*RNR21* (CNAG_01915) – left flanking primer 1 for CTR4
J1578	GCCACTCGAATCCTGCATGCTGTGTATGCGTCTAAGGGTT	*RNR21* (CNAG_01915) – left flanking primer 2 for CTR4
J1579	CGACAACGACTTCACCAATCATGGCTGCCGTCGAGACCCC	*RNR21* (CNAG_01915) – right flanking primer 1 for CTR4
J1580	TCGGAGATGGGTGAAGAGG	*RNR21* (CNAG_01915) – right flanking primer 2 for CTR4
J1581	TGTTCTCCCAGTCGTGAAG	*RNR21* (CNAG_01915) – probe primer for Southern blot
J1582	AGCCGTTCCAGATAGTTCC	*RNR22* (CNAG_02663) –5' screening primer for CTR4
J1583	CTTCGAGGATAACGCCTGC	*RNR22* (CNAG_02663) – left flanking primer 1 for CTR4
J1584	GCCACTCGAATCCTGCATGCGTTTGGCGTAGAAGAGTTGA	*RNR22* (CNAG_02663) – left flanking primer 2 for CTR4
J1585	CGACAACGACTTCACCAATCATGGCAACCCTACATCAAGC	*RNR22* (CNAG_02663) – right flanking primer 1 for CTR4
J1586	AAGCCACCAGTCTAGTTCG	*RNR22* (CNAG_02663) – right flanking primer 2 for CTR4
J1587	CCTGTTCCGTCATTCGGTC	*RNR22* (CNAG_02663) – probe primer for Southern blot
J1570	TGCACGATTTCGTCTGCTG	*RNR1* (CNAG_02208) –5' screening primer for H3
J1571	ACCCGATAACACAGGACAC	*RNR1* (CNAG_02208) – left flanking primer 1 for H3
J1643	CACTCGAATCCTGCATGCGAATCGTGGAAGAAGAGGGA	*RNR1* (CNAG_02208) – left flanking primer 2 for H3
J1644	ACCACAACACATCTATCACTCCCTCTTCTTCCACGATTC	*RNR1* (CNAG_02208) – right flanking primer 1 for H3
J1574	CCACGAGGAAGCATGATGAC	*RNR1* (CNAG_02208) – right flanking primer 2 for H3
J1576	TCGTCATCGTCCATTTCCTC	*RNR21* (CNAG_01915) –5' screening primer for H3
J1577	TGGAAGACTTTGCCTGGAC	*RNR21* (CNAG_01915) – left flanking primer 1 for H3
J1767	CACTCGAATCCTGCATGCTGTGTATGCGTCTAAGGGTT	*RNR21* (CNAG_01915) – left flanking primer 2 for H3
J1768	ACCACAACACATCTATCACATGGCTGCCGTCGAGACCCC	*RNR21* (CNAG_01915) – right flanking primer 1 for H3
J1580	TCGGAGATGGGTGAAGAGG	*RNR21* (CNAG_01915) – right flanking primer 2 for H3
J1582	AGCCGTTCCAGATAGTTCC	*RNR22* (CNAG_02663) –5' screening primer for H3
J1583	CTTCGAGGATAACGCCTGC	*RNR22* (CNAG_02663) – left flanking primer 1 for H3
J1611	GCCACTCGAATCCTGCATGCGTTTGGCGTAGAAGAGTTGA	*RNR22* (CNAG_02663) – left flanking primer 2 for H3
J1655	ACCACAACACATCTATCACATGGCAACCCTACATCAAGC	*RNR22* (CNAG_02663) – right flanking primer 1 for H3
J1642	TGCTCGGCGTACTGTTTGC	*RNR22* (CNAG_02663) – right flanking primer 2 for H3
J486	CTGGTTCCTATTCGTGCTTC	*SSN6* (CNAG_01902) –5' screening primer for deletion
J487	TGTTTACCCGTCTCTCTCG	*SSN6* (CNAG_01902) – left flanking primer 1 for deletion

J488	TCACTGGCCGTCGTTTTACGTCGGGTAAAGATACTTGGG	*SSN6* (CNAG_01902) – left flanking primer 2 for deletion
J489	CATGGTCATAGCTGTTTCCTGCTTCTGGGATGGAGATTGAC	*SSN6* (CNAG_01902) – right flanking primer 1 for deletion
J490	ATCTCCAGCGTAGCAGTGAC	*SSN6* (CNAG_01902) – right flanking primer 2 for deletion
J491	AAGGGTTATGCCTGAGAGC	*SSN6* (CNAG_01902) – probe primer for Southern blot
J497	AAGGAGTTTGAGAGGCTGG	*TUP1* (CNAG_02153) –5' screening primer for deletion
J498	TTTCCACAACACATCCGC	*TUP1* (CNAG_02153) – left flanking primer 1 for deletion
J499	TCACTGGCCGTCGTTTTACCCAACTGCTTTCTCATCTGAC	*TUP1* (CNAG_02153) – left flanking primer 2 for deletion
J500	CATGGTCATAGCTGTTTCCTGATCTTGCGAGTGGAAGTGG	*TUP1* (CNAG_02153) – right flanking primer 1 for deletion
J501	TCCAAAAAGTCAGCGACG	*TUP1* (CNAG_02153) – right flanking primer 2 for deletion
J502	GCAAGTTGAACACGCAAG	*TUP1* (CNAG_02153) – probe primer for Southern blot
J282	GCGGGTAGACATCAAATGC	*BDR1* (CNAG_02589) –5' screening primer for deletion
J283	TAGCGCCCAAACCAATCC	*BDR1* (CNAG_02589) – left flanking primer 1 for deletion
J270	TCACTGGCCGTCGTTTTACAGAAACTCTTCCACACGATG	*BDR1* (CNAG_02589) – left flanking primer 2 for deletion
J271	CATGGTCATAGCTGTTTCCTGCGTTCTACTGGGAATGATGG	*BDR1* (CNAG_02589) – right flanking primer 1 for deletion
J272	CATCAAACTCCTCAAACCC	*BDR1* (CNAG_02589) – right flanking primer 2 for deletion
J273	ATTCGTTGTTCACGCACG	*BDR1* (CNAG_02589) – probe primer for Southern blot
J1615	GATGACCCGGTTTCCCTTC	*RNR22* (CNAG_02663) –5' screening primer for deletion
J1616	TGGCAACCCTACATCAAGC	*RNR22* (CNAG_02663) – left flanking primer 1 for deletion
J1617	GCTCACTGGCCGTCGTTTTACAGTTCGGAAGGGCTGTGAG	*RNR22* (CNAG_02663) – left flanking primer 2 for deletion
J1618	CATGGTCATAGCTGTTTCCTGGAGATTGACAAGGCGGGTG	*RNR22* (CNAG_02663) – right flanking primer 1 for deletion
J1619	ACATGGCCGTGATGGGAAC	*RNR22* (CNAG_02663) – right flanking primer 2 for deletion
J1620	GCCTGTCGGCCACAAATTC	*RNR22* (CNAG_02663) – probe primer for Southern blot
